# Recommendations of the Polish Society of Diabetology and the Lifestyle of Patients with Type 2 Diabetes Mellitus: An Own Research

**DOI:** 10.3390/healthcare8040504

**Published:** 2020-11-20

**Authors:** Monika Gętek-Paszek, Beata Całyniuk, Alicja Ganczarek-Gamrot, Karolina Janion, Małgorzata Muc-Wierzgoń, Ewa Nowakowska-Zajdel

**Affiliations:** 1 Faculty of Health Sciences, College of Engineering and Health, 02-366 Warsaw, Poland; 2 Department of Human Nutrition, School of Health Sciences in Bytom, Medical University of Silesia in Katowice, 40-055 Katowice, Poland; bcalyniuk@sum.edu.pl; 3 University of Economics in Katowice, Faculty of Informatics and Communication, Department of Demography and Economic Statistics, 40-287 Katowice, Poland; alicja.ganczarek-gamrot@ue.katowice.pl; 4 Department of Nutrition-Related Disease Prevention, Department of Metabolic Disease Prevention, School of Health Sciences in Bytom, Medical University of Silesia in Katowice, 40-055 Katowice, Poland; karolina.janion@med.sum.edu.pl (K.J.); enowakowska-zajdel@sum.edu.pl (E.N.-Z.); 5 Department of Internal Medicine, School of Health Sciences in Bytom, Medical University of Silesia in Katowice, 40-055 Katowice, Poland; mwierzgon@sum.edu.pl; 6 Department of Clinical Oncology, No. 4 Provincial Specialist Hospital, 41-902 Bytom, Poland

**Keywords:** diet, smoking, physical activity, type 2 diabetes mellitus, lifestyle

## Abstract

Background and Objectives:Currently, diabetes is becoming a very serious challenge in medicine;type 2 diabetes mellitus (T2DM) is referred to as a noncontagious epidemic of the 21st century. The aim of the study is to assess the lifestyle of patients with type 2 diabetes, in particular eating habits, physical activity, and tobacco smoking, and to compare the obtained values with the latest recommendations of the Polish Society of Diabetology. Materials and Methods: The study group was comprised of 460 patients with type 2 diabetes, treated in the Diabetes Outpatient Clinic (*n* = 299) and the Clinical Department of Internal Medicine (*n* = 166). The study was conducted using a questionnaire, which included the Food Intake Frequency Questionnaire, 24 h dietary recall, and the International Physical Activity Questionnaire. Results: Abnormal excessive consumption of saturated fatty acids and salt and insufficient intake of dietary fiber was observed in the study group. Physical activity was insufficient in over 50% of the study group. In the study group, 17% of patients were smokers and the mean number of pack-years was 23. Conclusions:In the study group, excessive fat consumption (i.e.,saturated fatty acids) and dietary fiber deficiency were found in the daily diet.

## 1. Introduction

Currently, diabetes is becoming a very serious challenge in medicine;type 2 diabetes mellitus (T2DM) is referred to as a noncontagious epidemic of the 21st century. Above 80% of all T2DM cases worldwide occur in developed countries. According to the WHO, hypertension, tobacco smoking, and a high blood glucose level are the main risk factors for death. In 2015, 415 million adults (aged 20–79) were diagnosed with T2DM. In the same year, 5 million type-2 diabetes-related deaths were reported, which accounted for 14.5% of all deaths. About 50% of all such deaths were related to patients under 60 years of age [1].

According to the WHO Report (2016), the percentage of adults with T2DM increased from 4.7% to 8.5% between 1980 and 2014 [2].

Improper lifestyle, insufficient education and health prevention, and the aging of society are the main reasons for the constant increase in the number of new cases. In 2019, 59.3 million cases of adults (aged 20–79) with diabetes were reported in Europe. The number of deaths was estimated at 465,900 per year [1].

According to the International Diabetes Federation (IDF), there were 2344.6 million adults with T2DM in Poland in 2019, which accounted for 8.1% of this age group. It was also estimated that 990,000 individuals were undiagnosed [1].

The treatment of T2DM is not only based on maintaining target blood glucose levels but also involves normalizing body weight, blood pressure, and lipid profile. The current recommendations, including the recommendations of the Polish Society of Diabetology (PSD), indicate that the nutrition of patients with T2DM should not significantly differ from that of healthy individuals, however, individualization of nutritional recommendations is very important. The foundation of the individual approach contains guidelines for macro- and micronutrients, especially carbohydrates as a major macronutrient, which determine that periprandial insulin requirement should constitute 25–60% daily energy intake. Dietary fiber is essential, and its intake should be a minimum of 25 g per day or 15 g/1000 kcal. Additionally, fat composition is particularly important; saturated fats should provide less than 10% of the total calorie intake. Salt intake shouldnot exceed 5 g per day and should be more limitedfor patients with hypertension. Physical exercise is an integral part of proper comprehensive diabetes management, carbohydrate intake (% daily energy intake) is also determined by the level of physical activity. For optimal effects, exercise should be regular and undertaken at least every 2–3 days but preferably daily. Recommendations contain advice to quit smoking [3].

The aim of the study is to assess the lifestyle of patients with T2DM, in particular eating habits, physical activity, and tobacco smoking, and to compare the obtained values with the latest recommendations of the PSD.

## 2. Materials and Methods

### 2.1. Characteristics of the Study Group

The study group was comprised of 460 patients (262 women, 198 men) with T2DM. The subjects were treated at the Diabetes Outpatient Clinic (*n* = 299) and the Clinical Department of Internal Medicine, Bytom, Medical University of Silesia, Katowice, Poland (*n* = 166) and were enrolled according to the inclusion criteria.

Briefly, 262 women (57%) and 198 men (43%) were enrolled in the study (mean age: 66 years; SD: ±11.57). The mean duration of T2DM was 10 years (SD: ±7.53). Among women, the mean age and disease duration were 68 (SD: ±11.78) and 10 years (SD: ±8.23), respectively, and among men, 64 (SD: ±10.97) and 9 years (SD: ±6.45), respectively.

In addition, during treatment, patients were provided with basic nutrition information for lifestyle changes (information provided by doctors and nurses). Patients were not included in aneducational program or the care of a dietitian.

The characteristics of the study group are presented in Table 1.

In the study group, 44% of the respondents were obese and 34.5% were overweight. Among men, 1% were underweight, 27% had normal body weight, 30% were overweight, and 42% were obese. Among women, 2% were underweight, 14% had normal body weight, 38% were overweight, and 46% were obese. Mean body mass index (BMI) in the study group was 29.4 kg/m^2^ (SD: ±5.59); among women, it was 30.0 kg/m^2^ (SD: ±5.60), and for men, 29.0 kg/m^2^ (SD: ±5.55).

### 2.2. Study Design

The retrospective study was conducted using a questionnaire which consisted of the Intake Frequency Questionnaire (IFQ) prepared by The National Food and Nutrition Institute (NFNI) and The Institute of Cardiology [4], with the modification by Borawska et al. [5] as well as our own modification, the International Physical Activity Questionnaire (IPAQ), and medical record data.

Smoking-related questions included the number of years of active smoking, the mean number of cigarettes per day, and the period of nonsmoking for former smokers. The obtained data were converted into pack-years—the number of cigarette packs per day multiplied by the number of years of smoking—and used for further analysis.

The IFQ, with our modification, included 47 groups of food products, including white bread, wholemeal bread, confectioneries, flour dishes, cereal, rice, pasta, potatoes, raw vegetables, boiled vegetables, fruit, legume products, fish, poultry, giblets, sausages, deli meats, bacon, eggs, milk, fermented dairy products, cottage cheese, ripened and processed cheese, canned meat and fish, beef, pork, butter, soft/hard margarine, olive/rapeseed/sunflower oil, lard and pork fat, cake with cream, biscuits/shortbread biscuits, jam, honey, sweet drinks, sugar, sweeteners, wine, beer, vodka, salt, coffee, and tea. According to our modification, we added some products, the intake of which could influence metabolic parameters in patients with T2DM, e.g., salt. Some product groups (i.e., milk and milk beverages, meat, and margarines and oils) were also included.

The consumption of the above products had been assessed for 6 months prior to the study. The frequency of the consumption was assessed daily (1–2, 3–4, 5+ times), weekly (1, 2–3, 4–6 times), and monthly (0, 1–2, 3 times). Converting the obtained data to the daily energy value and nutrient intake was doneusing an algorithm prepared by the NFNI and the Institute of Cardiology (Table 2) [4]. “The photo album of products and dishes” was used to define portions of consumed products [6]. Using the “5D Diet” (NIFI, 2016, Poland) program, the values for consumed products, beverages, and meals were calculated as daily values. The obtained data were presented as SD, median, and minimum and maximum values.

### 2.3. Statistical Analysis

The data were then compiled and analyzed in Microsoft Excel 2010, and statistical calculations were performed in Statistica™ 10.0 (StatSoft, Kraków, Poland). The Shapiro–Wilk W-test, the Kolmogorov–Smirnov test, and Lilliefors’ test were applied to check if quantitative variables meet the normal distribution. The analysis was conducted using the Mann–Whitney U-test and the Kruskal–Wallis (ANOVA) test.

The statistical analysis included physical activity (MET), pack-years, energy value per day (kcal), average water intake (mL), protein total (g), animal protein (g), plant protein (g), fat (g), carbohydrates (g), saturated fatty acids (g), monounsaturated fatty acids (g), polyunsaturated fatty acids (g), cholesterol (mg), sucrose (g), dietary fiber (g), long-chain polyunsaturated fatty acids (mg), sodium (mg), vitamin D (ug), the percentage of daily energy from proteins (%), carbohydrates (%), fat (%), and alcohol (%).

For all analyses, the value of *p* < 0.05 was considered statistically significant.

### 2.4. Ethics Committee

The study was conducted in accordance with good clinical practice and the Declaration of Helsinki. The study received approval from the Medical University of Silesia’s Ethics Committee (Katowice, Poland; Resolution no. KNW/0022/KB433/13 of 18 June 2013). Participation in the study was voluntary.

## 3. Results

Based on the data from the IFQ, the mean daily energy intake was 1826 ± 522.11 kcal. The mean percentage of energy from protein was 18.5%, from carbohydrates 48%, from fat 33%, and from alcohol 0.5%.

The characteristics related to the intake of energy and the selected nutrients in the study group based on the IFQ are presented in Table 3.

Table 4 presents the results from the Mann–Whitney U-test and the Kruskal–Wallis (ANOVA) test, including sex, age, and sociodemographic features.

The results of the analysis showed statistically significant differences in terms of 14 ingredients (water, protein total, plant protein, animal protein, fat, carbohydrates, sodium, saturated fatty acids, polyunsaturated fatty acids, cholesterol, vitamin D, percent of energy from carbohydrates and alcohol) and energy from daily food intake in relation to gender.

Depending on the energy ratio and the selected nutrients, the difference was statistically significant for the majority of the variables under study. Men, on average, consumed more energy (W: 1708 ± 472 kcal, M: 1983 ± 545 kcal) and more protein (W: 83 ± 26 g; M: 93 ± 28 g), fats (W: 63 ± 23 g; M: 76 ± 30 g), carbohydrates (W: 227 ± 73 g/49 ± 8%; M: 253 ± 80 g/47 ± 8%), cholesterol (W: 240 ± 96 mg; M: 281 ± 123 mg), vitamin D (W: 2 ± 1 ug; M: 3 ± 2 ug), and water (W: 2517 ± 802 mL; M: 2614 ± 795 mL) compared to women.

The comparison of the mean results of the IFQ with the clinical recommendations of the PSD for T2DM patients is presented in Table 5.

The intake of saturated fatty acids (%), dietary fiber (g), and sodium (mg) was not consistent with the recommendations of PSD. The *2019 PSD Clinical Recommendations* suggest thatenergy from carbohydrates should be at the level of 45% (25–60%) of energy from the daily food intake, with possible modifications depending on physical activity and comorbidities. The glycemic index (GI) of products should be <55 [3].

Insufficient physical activity was observed in 50% of the study group, sufficient activity was noted in 44%, and high activity was found in 6% of patients based on the division of physical activity into categories (insufficient <600 MET min/week, sufficient between 600–3000 MET min/week, and high >3000 MET min/week), according to the criteria described in the Polish version of the IPAQ.

In the study group, the mean level of physical activity (MET min/week) was 978 ± 1146.36 MET, and the median was 660 MET. A MET scoreof 0 was found in some hospitalized patients at the time of the assessment.

The results of the Kruskal–Wallis (ANOVA) test showed that physical activity in the study group was different depending on age, education, professional activity, and the place of residence (Table 6).

In the group of patients ≥65 years of age, the level of physical activity was statistically significantly lower compared to the group of younger patients. Patients with vocational education were characterized by statistically significantly higher physical activity compared to other groups, whereas the lowest physical activity was observed among those with primary and higher education.

In the study group, 79 patients reported active smoking (W: 30, M: 49), which accounted for 17% of the study group. Nonsmokers accounted for 83% (*n* = 381), including former smokers (*n* = 168). Smokers <65 years of age constituted a group of 46 patients; 33 subjects aged ≥65 were active smokers.

In addition, statistically significant differences were observed between the BMI of smokers and nonsmokers (*p* = 0.0033). Smokers had a lower BMI. Statistical analysis also showed that BMI was statistically lower in the group aged ≥65 (*p* = 0.0117).

The mean number of packs in the group of active smokers (*n* = 79) was 23.05, whereas in the group of former smokers, it was 21.82.

## 4. Discussion

An abnormal BMI is very common in type 2 diabetic patients, as confirmed by a number of studies. Reducing excessive body weight and compliance with dietary recommendations by patients with T2DM is an essential component of therapy. A well-composed diet has positive effects on patient health by promoting the maintenance of metabolic control and preventing and treating chronic complications of diabetes.

The recommendations of PSD constitute advice to eat frequent, regular meals [3]. In the present analysis, the study group mostly consumed 3 meals daily (40%), with the mean number of meals being 4. The group that consumed 4 or 5 meals accounted for 28.2% and 21.74%, respectively. In another Polish study (*n* = 328, 64.2 ± 7.7 years) conducted among patients with T2DM, the largest group (38%) of patients consumed 5 meals daily [7]. In the Wrocław (acity in Poland) study conducted among 75 patients with T2DM, the majority consumed 4 meals (64.7% of women and 38.9% of men) [8]. In the study among elderly patients with T2DM, the largest group (76.2%) consumed 4 to 5 meals per day [9]. Differences in study results may be due to the fact that the above reports were conducted among participants of training related to diabetes management, which may indicate their higher health awareness compared to our study group [7].

In T2DM, the major goals are not only to maintain good metabolic control of the disease but also to reduce excess body weight and maintain the desired body weight. When planning diets, individual patient nutritional and cultural preferences, age, gender, level of physical activity, and economic status should be taken into account. Proper energy and macronutrient intakesare significant inproviding therapy and prevention [3]. Based on the results of the analysis obtained from the IFQ, the mean energy intake and the selected nutrients were determined. The mean energy of the daily food intake according to the IFQ was 1822.13 ± 522.11 kcal; the percentage of energy derived from carbohydrates was 48.3% ± 7.95%, from protein 18.48% ± 4.04%, and from fat 32.7% ± 7.45%. The results of a survey among 1523 Algerians (58.7 ± 9.9 years) using the diet questionnaire suggested the mean energy consumption of 2668 ± 320 kcal; 55% ± 10% of energy was derived from carbohydrates, 13% ± 3% from protein, and 30% ± 7% from fats. The mean intake of dietary fiber was 26 ± 3 g, while in the present study, it was 16.94 ± 5.93 g. In the above study, 64% of subjects were obese compared to 44% in the present study, which may be related to differences in mean energy [10]. In a Jordan study on 198 patients with T2DM (55.9 ± 9.3 years), the mean daily energy consumption was 2279.2 ± 944.4 kcal; 56.7% ± 7.5% of energy was derived from carbohydrates, 15.9% ± 3.1% from protein, and 27.7% ± 6.5% from fat. The data were obtainedfrom the consumption frequency questionnaire. The mean BMI in that group was 32.8 ± 5.9 kg/m^2^, while in the present study, it was 29.43 ± 5.59 kg/m^2^ [11]. Based on the frequency questionnaire, a Japanese study on 260 patients with T2DM (65.7 ± 9.3 years) showed a mean daily energy consumption of 1821.5 ± 400.1 kcal. Energy derived from carbohydrates accounted for 56.9% ± 7.7% of daily mean energy, protein 15.3% ± 2.4%, and fat 26.9% ± 6.4%. Despite the similar mean daily energy from food intake in the above report and the present study, mean BMI values were different. In the Japanese group, the mean BMI was 24.0 ± 4.2 kg/m^2^, whereas in the present study, it was 29.43 ± 5.59 kg/m^2^ [12].

It was shown that the energy from daily food intake and the majority of nutrients included in the analysis were statistically significantly different, depending on gender. This is related to the energy value and percentage of energy derived from carbohydrates. The mean energy consumption was higher in the group of men (*n* = 198) at 1981.64 kcal, while in the group of women (*n* = 262), it was 1707.56 kcal. The percentage of energy derived from carbohydrates was higher in the group of women (49.0%) compared to the group of men (47.4%). In a Chinese study, the mean value of energy in the group of men (*n* = 345; 55.73 ± 12.73) was 2027.28 ± 598.93 kcal, whereas in the group of women (*n* = 262; 57.27 ± 11.64), it was 1703.93 ± 485.2 kcal. The difference was statistically significant, as was the percentage of energy from individual macronutrients. In the group of women, the percentage of energy from carbohydrates and protein was higher, and the percentage of energy from fat was lower in daily food intake [13].

Physical exercise is an integral part of proper comprehensive diabetes management. For optimal effects, exercise should be regular and undertaken at least every 2–3 days but preferably daily [3]. Based on the short version of the IPAQ, physical activity (MET min/week) was assessed in the study group. Fifty percent of the group (*n* = 228) were patients with insufficient physical activity (<600 MET), 44% of patients with 600–3000 MET, and 6% of subjects with high physical activity (>3000 MET). Some hospitalized patients did not have any physical activity (0 MET). In a Brazilian study on 200 patients with T2DM (50 subjects from the control group; aged 40–60), the influence of physical activity on the quality of life was assessed. Data on physical activity were collected using the IPAQ. Among patients with T2DM, 40% had a sedentary lifestyle, 33% were characterized by irregular physical activity, 20% were active, and 8% very active [14]. In a Turkish study on patients with insulin resistance (*n* = 147; aged 45 ± 12.25) and a control group without insulin resistance (*n* = 147; aged 41.39 ± 10.32), all patients were overweight or obese. Among the group with insulin resistance, the mean level of physical activity (IPAQ) was 538.71 ± 382.81 MET [15]. In the present study on patients with T2DM, physical activity (MET) was, on average, 977.53 ± 1136.36 MET. In a Malaysian study on 204 patients with T2DM (61.3 ± 10.3 years), 62.7% did not have any physical activity [16]. When the present study was compared to the above reports, in which only one study used the IPAQ, similarities, the tendency to low physical activity among patients with T2DM or insulin resistance was observed.

Patient management should include therapeutic lifestyle changes encompassing, especially, a balanced diet, regular physical activity, and avoidance of tobacco smoking [3]. As a result of the analysis, it was shown that 17% (*n* = 79) of patients were smokers and 83% (*n* = 381) were nonsmokers, of whom 168 were former smokers. In the Wrocław study (*n* = 75), 11.8% of women and 16.7% of men were active smokers [8]. In a Sino-British study, in the British group (*n* = 1172), 15.7% were active smokers;however, no data related to the Chinese group were provided [17]. In the Leader-4 trial, current and former smokers were enrolled in one group;they accounted for 58.55% of the whole group (*n* = 9340).In the group from Europe (Czech Republic, Poland, Romania, Austria, Belgium, Denmark, Finland, France, Germany, UK, Greece, Ireland, Italy, The Netherlands, Spain, Sweden, Turkey, and Serbia; *n* = 3521),active smokers accounted for 66.1%.In the group from North American countries (USA and Canada; *n* = 2821), they accounted for 63.5%, and in the group from other countries (India, China, South Korea, Taiwan, South Africa, Australia, Brazil, Mexico, Arab Emirates, Russia, and Israel; *n*  =  2998), they accounted for 45% [18]. In the present study, a group of former and current smokers accounted for 53.7%. In a Spanish study on 403 patients with T2DM, 13.6% were active smokers, 33.7% were former smokers, and 52.6% had never smoked [19]. In a Polish study on 106 patients with T2DM (86% aged > 50), 14% were active smokers, 44% were former smokers, and 41% had never smoked [20].

The diet of the studied patients was improperly balanced. Low physical activity and the use of stimulants contributed to the formation of metabolic disorders, including incorrect body weight. The authors’ clinical experience shows that patients with T2DM continue to make similar nutritional mistakes.

Currently, we can also observe a growing trend to manage very low carbohydrate or high-fat diets to treat T2DM or insulin resistance. These diets, most often based on animal products, provide too much saturated fatty acids, the consumption of which should be limited to a maximum of 10% from daily energy intake. There are the results of intervention studies, which achieved the expected metabolic effects after the use of such diets but were planned and took into account plant sources of protein and fats or assumed the participation of mainly unsaturated fatty acids and low saturated fatty acids. Due to complications in the form of renal diseases, patients with T2MD who are also taking SGLT-2 inhibitors are not recommended to consume high-fat diets. There areno well-designed long-term studies on this topic [21]. PSD recommends various healthy and tested diet patterns, i.e.,the DASH diet, the Mediterranean diet, and plant-based diets, withmodifications for each individual [3].

This situation may indicate insufficient educational activities. PSD and other international societies should undertake educational activities that take into account the current nutritional needs of patients.

In addition, education related to nutrition, physical activity, and smoking should be provided, especially to young patients with T2DM, regardless of gender. Considering that a number of individuals are still undiagnosed with diabetes, such education should be applied to all adults who visit primary care physicians, especially those who are overweight and obese.

With reference to other research conducted in Poland and around the world, and the results obtained in this study, we are additionally convinced that educational activities should be individualized not only to gender, age, education level, and work performed but also tothe place of residence, taking into account the relevant tradition and culture of nutrition and lifestyle.

## 5. Conclusions

Based on the results of their own research, the authors noticed dietary mistakes and a strong need to correct them by educating patients. It is necessary to individualize dietary and lifestyle recommendations depending on many factors, including age, nutritional status, comorbidities, social status, or cultural factors, i.e., the dietary habits of the country. Assessment of adherence to recommendations on nutrition and physical activity should be carried out systematically. The interdisciplinary team (doctors, nurses, dieticians, physiotherapists) can then take effective action, leading to an improvement in the quality of life and overall survival, decreasing the risk of complications in patients with metabolic diseases.

## 6. Limitations

In this study, the group of patients was diverse (i.e., age, comorbidities, outpatient vs. hospitalized patients). The authors suggest extending the research to include the assessment of compliance with PSD recommendations by younger age groups. They propose the inclusion of new anthropometric indicators as well as body composition analysis in future research.

## Figures and Tables

**Table 1:** Characteristics of the study group, including gender and age.

Features		Women (*n* = 262)		Men (*n* = 198)		Age<65 (*n* = 196)		Age≥65 (*n* = 264)		Total (*n* = 460)	
		*n*	*%*	*n*	*%*	*n*	*%*	*n*	*%*	*n*	*%*
Place of residence	City	254	55.0	191	42.0	188	41.0	257	56.0	445	97.0
	Country	8	2.0	7	2.0	8	2.0	7	2.0	15	3.0
Marital status	Single	32	7.0	30	7.0	31	7.0	31	7.0	62	13.0
	In a relationship	149	32.0	131	28.0	128	28.0	152	33.0	280	61.0
	Widow/widower	81	18.0	37	8.0	37	8.0	81	18.0	118	26.0
Education	Primary	85	18.0	37	8.0	52	11.0	70	15.0	122	27.0
	Vocational	87	19.0	88	19.0	73	16.0	102	22.0	175	38.0
	Secondary	73	16.0	53	12.0	52	11.0	74	16.0	126	27.0
	Higher	17	4.0	20	4.0	19	4.0	18	4.0	37	8.0
Professional status	Unemployment/retirement	220	48.0	148	32.0	132	29.0	236	51.0	368	80.0
	White-collar workers	24	5.0	20	4.0	26	6.0	18	4.0	44	10.0
	White- and blue-collar workers	18	4.0	30	7.0	38	8.0	10	2.0	48	10.0

**Table 2:** Categories of intake frequency [4].

Intake Frequency from the IFQ	Intake Frequency per Day	Intake Frequency per Month (30 Days) [4]
Daily	1	30
4–6 times per week	0.71	21.4
2–3 times per week	0.69	20.7
Once per week	0.14	4.3
3 times per month	0.1	3.0
1–2 times per month	0.05	1.5
Rarely	0	0

IFQ: Intake Frequency Questionnaire.

**Table 3:** Energy and selected nutrients intake (per day) based on the IFQ in the study group.

Energy Value and Nutrients	Mean	Median	Minimum	Maximum	SD
Energy value (kcal)	1826.13	1704.76	632.03	4201.49	±522.11
Water (mL)	2517.21	2454.24	290.84	5194.74	±802.37
Protein total (g)	87.25	84.86	26.99	218.20	±27.52
Animal protein (g)	59.30	57.60	9.63	165.26	±23.77
Plant protein (g)	27.88	27.22	4.16	58.67	±8.69
Fat (g)	68.89	64.76	12.62	198.14	±27.08
Carbohydrate (g)	237.98	220.37	62.86	571,52	±77.07
Sodium (mg)	5257.44	5012.98	769.29	12,108.40	±2024.23
Saturated fatty acids (g)	22.92	21.26	2.19	63.35	±10.38
Monounsaturated fatty acids (g)	28.24	26.75	4.13	68.24	±11.81
Polyunsaturated fatty acids (g)	15.10	13.38	1.97	77.02	±8.04
Cholesterol (mg)	257.45	250.96	24.20	747.35	±110.08
Sucrose(g)	22.23	15.78	3.00	132.36	±19.27
Dietary fiber (g)	16.94	16.47	5.00	38.67	±5.93
Vitamin D (µg)	2.40	2.21	0.03	10.71	±1.63
Long-chain polyunsaturated fatty acids (g)	0.13	0.07	0.00	1.56	±0.24
Protein-derived energy (%)	18.48	18.04	6.86	35.02	±4.04
Saturated fatty acids (%)	11.33	11.26	3.12	23.08	±3.50
Monounsaturated fatty acids (%)	13.93	13.85	3.85	26.44	±3.90
Polyunsaturated fatty acids (%)	7.26	6.85	1.84	23.38	±2.63
Fat-derived energy (%)	32.70	32.54	13.77	60.43	±7.45
Carbohydrate-derived energy (%)	48.31	48.68	17.20	71.04	±7.95
Alcohol-derived energy (%)	0.52	0.00	0.00	12.85	±1.62

**Table 4:** Comparative analysis between the selected nutrients depending on sex, age, and sociodemographic factors.

Energy Value and Nutrients	Sex	Age	Education	Professional Activity	Marital Status	Place of Residence
Energy value (kcal)	**<0.01**	0.09	0.57	0.15	0.51	0.39
Water (mL)	**0.02**	0.54	0.82	0.74	0.14	0.38
Protein total (g)	**<0.01**	0.79	0.65	0.78	0.82	0.37
Animal protein (g)	**<0.01**	0.52	0.20	0.80	0.63	0.63
Plant protein (g)	**<0.01**	0.31	0.46	0.48	0.95	0.12
Fat (g)	**<0.01**	0.05	0.12	0.09	0.36	0.79
Carbohydrates in total(g)	**<0.01**	0.12	0.45	0.09	0.99	0.12
Sodium (mg)	**<0.01**	0.37	0.47	0.16	0.80	0.52
Saturated fatty acids (g)	**<0.01**	**<0.01**	0.06	0.05	0.21	0.82
Monounsaturated fatty acids (g)	**<0.01**	0.30	0.14	0.27	0.29	0.76
Polyunsaturated fatty acids (g)	**<0.01**	0.41	0.63	0.07	0.06	0.41
Cholesterol (mg)	**<0.01**	0.52	0.18	0.94	0.65	0.96
Sucrose (g)	0.87	0.23	0.74	0.56	0.46	0.65
Dietary fiber (g)	0.71	0.28	0.36	0.30	0.97	0.07
Vitamin D (µg)	**<0.01**	**0.03**	0.75	0.38	0.93	0.51
Long-chain polyunsaturated fatty acids (g)	0.32	0.16	0.14	0.28	0.61	0.76
Protein-derived energy (%)	0.84	0.16	0.31	0.74	0.86	0.82
Saturated fatty acids (%)	0.34	**0.01**	0.10	0.42	0.14	0.28
Monounsaturated fatty acids (%)	0.18	0.54	0.28	0.72	0.16	0.63
Polyunsaturated fatty acids (%)	0.75	0.83	0.87	0.49	0.06	0.88
Fat-derived energy(%)	0.44	0.52	0.41	0.70	0.55	0.56
Carbohydrate-derived energy (%)	**0.03**	0.83	0.08	0.93	0.90	0.50
Alcohol-derived energy (%)	**<0.01**	0.54	0.57	0.12	0.58	0.36

Statistically significant results are bolded.

**Table 5:** The comparison between the 2019 recommendations of the PSD and the data from the IFQ [3].

Energy Value and Nutrients	Recommendations of the PSD	Data from the IFQ (*n* = 460)
Energy value (kcal)	2051.00 ± 500.00	1826.13
Protein total (%)	15–20	18.48
Fat (%)	25–40	32.70
Saturated fatty acids (%)	<10	**11.33**
Monounsaturated fatty acids (%)	up to 20	13.93
Polyunsaturated fatty acids (%)	6–10	7.26
Cholesterol (mg)	<300.00	257.45
Carbohydrates (%)	~45 (25–60)	48.31
Dietary fiber (g or g/1000 kcal)	≥25 or 15 g/1000 kcal	**16.94 or 9.3 g/1000 kcal**
Salt (mg)	<5000.0	**13,143.6**

PSD: Polish Society of Diabetology;IFQ: Intake Frequency Questionnaire; Values not complying with the PSD recommendations are bolded.

**Table 6:** Comparative analysis of physical activity (MET) depending on sex, age, and sociodemographic factors.

**Physical Activity (MET)**	**Sex**	**Age**	**Education**	**Professional Activity**	**Marital Status**	**Place of Residence**
	0.61	0.03	0.02	<0.01	0.39	<0.01
